# "Hypoxia-induced down-regulation of microRNA-449a/b impairs control over targeted SERPINE1 (PAI-1) mRNA - a mechanism involved in SERPINE1 (PAI-1) overexpression"

**DOI:** 10.1186/1479-5876-8-33

**Published:** 2010-04-01

**Authors:** Michaela Muth, Katharina Theophile, Kais Hussein, Christoph Jacobi, Hans Kreipe, Oliver Bock

**Affiliations:** 1 Institute of Pathology, Hannover Medical School, Carl-Neuberg-Strasse 1, 30625 Hannover, Germany; 2 Department of Pediatric Nephrology, Hannover Medical School, Carl-Neuberg-Strasse 1, 30625 Hannover, Germany

## Abstract

**Background:**

In damaged organs tissue repair and replacement of cells by connective tissue provokes a response of fibroblasts to cellular stress factors such as hypoxia.

MicroRNAs (miRNA) are small non-coding RNA molecules which bind to their mRNA targets which eventually lead to repression of translation. Whether the response of fibroblasts to stress factors also involves the miRNA system is largely unknown.

**Results:**

By miRNA profiling we identified down-regulation of miRNA-449a/b expression in hypoxic fibroblasts. Specific miRNA inhibitors and mimics showed direct evidence for targeting the serine protease inhibitor (serpin) protein (SERPINE1; plasminogen activator inhibitor-1, PAI-1) by miRNA-449a/b leading to SERPINE1 mRNA and protein up- and down-regulation, respectively. SERPINE1 expression *in vivo *could be located predominantly in areas of fibrosis and remodeling.

**Conclusions:**

Our study offers serious lines of evidence for a novel hypoxia-dependent mechanism involving hypoxia-induced decrease of clustered miRNA-449a/b, hypoxia-induced amplification of concomitant increase of targeted SERPINE1 (PAI-1) and its overexpression in tissues showing a hypoxic environment.

## Background

Extracellular matrix (ECM) deposition and fibrotic remodeling in damaged tissues is the principle to regain integrity when affected tissues have no or only limited capability for self-renewal [1]. Damage of tissues and cells can be induced by hypoxia, infections and chronic irritants but has in common that the response to injury is very similar. Mechanisms of reparation will take place after trivial wounding of the skin but are also demonstrable in organ transplantation when the allograft is chronically rejected and compartments lose, step by step, their function due to accumulation of ECM.

While hypoxia can cause damage and tissue destruction, the process of tissue repair and remodeling is also accompanied by low oxygen levels in certain areas causing a stress response of fibroblasts and other cell types involved in tissue remodeling [2].

Tissue damage leads to accumulation of cytokines and numerous growth factors like members of the transforming growth factor β family (TGF β 1-3) and bone morphogenetic proteins (BMPs) [3-5]. Another set of tissue remodeling factors belong to the family of matrix-metalloproteinases (MMPs) and their inhibitors TIMPs (tissue inhibitors of metalloproteinases), [6,7]. Comparable with MMPs is the action of plasmin and members of the plasminogen activator urokinase-type (uPA) and -tissue-type (tPA) which mediate proteolysis of ECM components but also activation of latent, inactive MMPs [8,9]. Their negative regulator serine protease inhibitor (serpin) protein (SERPINE1; plasminogen activator inhibitor-1, PAI-1) is an inhibitor of plasmin action and was shown to be a target of TGFβ-1 which implicates cross-talks between the members of the pro-fibrotic system [10,11].

The microRNA (miRNA) system controls the fate of mRNA molecules and stands for an important cellular regulatory mechanism. Post-transcriptional regulation of mRNA by the microRNA (miRNA) system is thereby highly conserved among species, including humans, and expression of hundreds of miRNA in tissues and cellular lineages has already been reported [12]. Transcripts of miRNA genes form ~ 100 nucleotide-long hairpin primary miRNA (pri-miRNA) precursors which are subsequently translocated from the nucleus to the cytoplasm. They are further processed to shorter double-stranded premature miRNA (~ 80 nucleotide-long pre-miRNA) and finally to mature/functionally active ~ 20 nucleotide-short miRNA species [13]. A given biologically active miRNA will then be incorporated into the so-called RNA-induced silencing complex (RISC), where binding of mRNA targets by miRNA takes place. Semi-complementary binding of miRNA to the 3'-untranslated region (3'-UTR) of target mRNA results in translational suppression or cleavage of mRNA, respectively [13]. Due to these semi-complementary miRNA-mRNA binding abilities, one miRNA might control several potential mRNA targets and, vice versa, one mRNA might be suppressed by several miRNA species [12].

One particular miRNA was recently shown to be involved in tissue remodeling and matrix deposition, i.e. exclusive up-regulation of miRNA-21 by fibroblasts was shown to be involved in myocardial fibrosis [14]. The up-regulation of miRNA-21 was suggested to be stress induced what attracted our attention because the stress response of fibroblasts on the miRNA level has yet not been accomplished.

We therefore investigated primary fibroblasts under hypoxic stress, determined the miRNA profile, and screened for designated mRNA targets involved in tissue remodeling and fibrosis development. Lastly, we investigated tissues from kidney transplants showing chronic remodeling for aberrations in miRNA expression and impact on designated SERPINE1 mRNA and protein fate.

## Methods

### Cell lines and culture

Primary human fibroblasts (F-18, dermal origin, kindly provided by Dr. Miriam Wittmann, Faculty of Biological Sciences, University of Leeds, UK) and M15D [15] were cultured as monolayers in RPMI 1640 containing 10% fetal calf serum (FCS) and 1% antibiotics until subconfluence. Metaphase cytogenetics showed no evidence for clonal aberrations in these primary cell lines.

### Hypoxia in cell culture

Cell culture flasks were placed into anaerobic jars for 24 hours (Anaeropack for cell culture, Mitsubishi Gas Chemicals, Tokyo, Japan) to induce hypoxic culture conditions as described [16,17]. Briefly, the Anaeropack for Cell contains sodium ascorbate as the principal ingredient which absorbs oxygen and generates carbon dioxide by oxidative degradation. Magnesium hydroxide is used as a scavenger for carbon dioxide. These reagents are located in paper sachets and are placed into the jars. Controls were cultured in parallel under normal oxygen concentration of ~ 20%. Viability tests of cells in culture were performed before and after 24 hours by Trypan blue exclusion.

### Transfection of fibroblasts with miRNA-449 inhibitors and miRNA-449 mimics

The HiPerfect Transfection Reagent (# 301705, Qiagen, Hilden, Germany) was used for transfection with anti-hsa-miRNA-449a/b Inhibitor (#MIN0001541, target sequence UGG CAG UGU AUU GUU AGC UGG U; #MIN0003327, target sequence AGG CAG UGU AUU GUU AGC UGG C; respectively, both Qiagen, Hilden, Germany) or with Syn-hsa-miRNA-449a/b Mimic (#MSY0001541, target sequence UGG CAG UGU AUU GUU AGC UGG U; #MSY0003327, target sequence AGG CAG UGU AUU GUU AGC UGG C; respectively, both Qiagen, Hilden, Germany). A negative control siRNA (#1027280; Qiagen, Hilden, Germany) was transfected as well. This control has no homology to any mammalian gene. Additionally, primary fibroblasts were treated with the HiPerfect Transfection Reagent only.

Transfection was performed according to the manufacturer's protocol for adherent cells in 6-well plates with 2 different concentrations as follows: miRNA inhibitor 50 nM and 100 nM; miRNA mimic 5 nM and 10 nM, respectively. After transfection cell culture flasks were placed into anaerobic jars for 24 hours. Controls were cultured in parallel under normal oxygen tension.

### Human tissue specimens

From the archive of the Institute of Pathology, Hannover Medical School, we retrieved formalin-fixed, paraffin-embedded (FFPE) tissues and biopsies from kidney transplants (n = 5) showing proven histopathological features of chronic allograft nephropathy with interstitial fibrosis, glomerular and tubular atrophy accompanied by only a mild chronic inflammation. Control samples (n = 4): 2 different tissue specimens from 1 kidney transplant immediately explanted due to allograft-steal syndrome, 3 tissue specimens taken from 3 tumor nephrectomies due to renal cell carcinoma. These control specimens showed normal kidney morphology and no evidence for involvement by carcinoma. Relevant clinical and morphological information are listed in Table 1.

**Table 1:** Condensed data on patients histories and sample type

Gender	Age	Underlying disease	Year of Tx.	Kidney explant or biopsy	Why indicated ?	Histo- pathological diagnosis	Tissue samples analyzed n =	**Symbol**^**#**^**in Figure **8
female	60	Systemic Lupus erythematosus	1999*	Kidney explant (01/2007)	Acute renal failure	(Interstitial fibrosis, tubular atrophy) Banff 2007, 5. II [34]	2	Black circle Black square

female	41	Systemic vasculitis (Purpura Schoenlein-Henoch)	1996	Kidney explant (01/2007)	Acute renal failure	(Interstitial fibrosis, tubular atrophy) Banff 2007, 5. II [34]	1	Black triangle

male	17	Obstructive uropathy	2006*	Kidney explant (08/2007)	Impaired kidney function	(Interstitial fibrosis, tubular atrophy) Banff 2007, 5. III [34]	2	Black reverse triangle Black rhomb

female	45	Chronic glomerulo-nephritis	1996	Indication biopsy (04/2007)	Impaired kidney function	Chronic tubulo-interstitial injury, evidence for calcineurin inhibitor toxicity (CNIT)	1	White circle

male	72	Chronic interstitial nephritis	2007	Indication biopsy (06/2007)	Impaired kidney function	Interstitial fibrosis, tubular atrophy, vasculopathy) Banff 2007, 5. II [34] **	1	White square

female	42	Systemic Lupus erythematosus	2005	Kidney explant (2005)	Allograft-steal-syndrome	Diffuse tubular damage	2	Black circles in plot "Control"
	
Not known (anonymized)		RCC	n.a.	Tumor nephrectomy	RCC	Normal	1	
								
		RCC			RCC		1	
								
		RCC			RCC		1	

2^nd ^allograft in patients history, ** = "old-for-old" kidney allocation, Tx. = transplantation, RCC = renal cell carcinoma, n.a. = not attributable. ^#^Two symbols per case mean that 2 different tissue blocks were analyzed in the same patient.

### RNA extraction

The monolayer of 1 culture flask (75 cm^2^) was suspended in 1 ml TRIZOL Reagent (Invitrogen, Carlsbad, CA, USA) and stored over night at -20°C. The extraction of total RNA was accomplished as instructed by the manufacturer. Total RNA was extracted from FFPE tissues following guanidinium isothiocyanate/Proteinase K-based digestion, and conventional organic extraction using phenol/chloroform as we previously described [18].

### cDNA synthesis

The TaqMan MicroRNA Reverse Transcription Kit (Part No. 4366596, Applied Biosystems, Foster City, CA, USA) and the Megaplex™ RT Primer A (Megaplex™ RT Primers, Human Pool A, Part No. 4399966, Applied Biosystems, Foster City, CA, USA) were used to synthesize complimentary DNA (cDNA) for the TaqMan MicroRNA Array (TaqMan^® ^Human MicroRNA A Array v2.0, Part No. 4398965, Applied Biosystems, Foster City, CA, USA). The final reverse transcription reaction consisted of 3.0 μL 500 ng total RNA and 4.5 μL RT reaction mix. cDNA was synthesized as described in the manual of Run Megaplex™ Pools without pre-amplification (Applied Biosystems, Foster City, CA, USA).

The TaqMan MicroRNA Reverse Transcription Kit (Part No. 4366596, Applied Biosystems, Foster City, CA, USA) was also used to synthesize cDNA for the individual TaqMan MicroRNA Assays (RNU48 Assay ID 001006, RNU49 Assay ID 001005, hsa-miRNA-449a Assay ID 001030, hsa-miRNA-449b Assay ID 001608, Applied Biosystems, Foster City, CA, USA) by using 0.01 μg total RNA. For real-time RT-PCR on the mRNA level the High Capacity cDNA Reverse Transcription Kit (Part No. 4368814, Applied Biosystems, Foster City, CA, USA) was applied by using 1 μg total RNA.

### MicroRNA profiling

The TaqMan Human MicroRNA A Array v2.0 represents a 384-well format (Part No. 4398965, Applied Biosystems, Foster City, CA, USA). 377 human miRNAs, three endogenous controls and one negative control were included. The array was loaded with a mixture of 450 μL TaqMan Universal PCR Master Mix, No AmpErase UNG (Part No. 4324018, Applied Biosystems, Foster City, CA, USA), 6 μL Megaplex RT product and 444 μL HPLC-H_2_O. The TaqMan Human MicroRNA A Array was performed on a 7900HT Fast Real-Time PCR system and was recorded by the 7900HT SDS 2.3 software (Applied Biosystems, Foster City, CA, USA). Only miRNA showing at least 3-fold increase or decrease were included in the results section. LDA data of miRNA expression are shown in the additional file 1.

### Gene expression profiling by custom made low density arrays

A set of 45 genes related to tissue remodeling and fibrosis and 2 reference genes (*RNA-polymerase 2 *- *POLR2A *and *β-Glucuronidase - GUSB*) was selected for custom made TaqMan Low Density Arrays (LDA; Applied Biosystems, Foster City, CA, USA). Details of the genes spotted on the LDA are shown in the additional file 2. The reference gene *Glyceraldehyde-3-phosphate dehydrogenase *(*GAPDH*) was declared to be mandatory on LDA according to the distributor but due to the well known up-regulation of *GAPDH *by hypoxia it was not considered as a reference gene for subsequent relative quantification. The entire gene set was spotted 8-fold (8 × 48) on the 384-well micro fluidic card allowing concomitant investigation of 8 samples per run. The array was loaded with a mixture of 5 μL cDNA, 45 μL HPLC-H_2_O (J.T. Baker, Phillipsburg, NJ, USA) and 50 μL Universal PCR Master Mix (Part No. 4352042, Applied Biosystems, Foster City, CA, USA). TaqMan low density arrays were performed on a 7900HT Fast Real-Time PCR system and recorded by the 7900HT SDS 2.3 software (Applied Biosystems, Foster City, CA, USA). Only genes showing at least 3-fold up- or down-regulation were included in the results section. LDA data of mRNA expression are shown in the additional file 1.

### Re-evaluation of miRNA and mRNA targets

The expression of hsa-miRNA-449a (ID 001030) and hsa-miRNA-449b (ID 001608) along with reference small RNA molecules RNU48 (ID 001006) and RNU49 (ID 001005) was re-evaluated by real-time PCR (TaqMan 7500 Fast Real-Time PCR system, Applied Biosystems). Each reaction consisted of 10 μL TaqMan Universal PCR Master Mix, No AmpErase UNG (Part No. 4324018, Applied Biosystems, Foster City, CA, USA), 1 μL of the invidual TaqMan MicroRNA Assay (Applied Biosystems, Foster City, CA, USA), 4 μL HPLC-H_2_O (J.T. Baker, Phillipsburg, NJ, USA) and 5 μL cDNA.

The re-evaluation of *SERPINE1 *mRNA expression was performed with 2 different assays (Hs01126606_m1, Hs00167155_m1, respectively, Applied Biosystems, Foster City, CA, USA) in addition to *POLR2A *(Hs00172187_m1) and *GUSB *(Hs99999908_m1) as reference genes in 20 μL reactions containing 10 μL TaqMan Gene Expression Master Mix, 1 μL of the individual TaqMan Gene Expression Assay (both Applied Biosystems), 8 μL pure water, and 1 μL cDNA.

### Quantification of real-time PCR data

Recorded C_T _values were converted into ΔC_T _values relative to reference genes *POLR2A *and *GUSB *for mRNA and *RNU48 *and *RNU49 *for microRNA, respectively. The 2^-ΔΔCT ^method [19] was applied by using Excel 8.0 (Microsoft, Redmond, WA, USA). Results were statistically analyzed and graphically visualized with Prism 5.0 (GraphPad Software, San Diego, CA, USA) by applying the one-way analysis of variance (ANOVA) test followed by Tukey's post-test.

### Immunocytochemistry and immunohistochemistry

F-18 and M15D were transfected as described and cultured as monolayers on 4-well chamber slides (Nalge Nunc, Naperville, IL, USA) under hypoxia for 24 hours. Primary human fibroblasts were treated with the HiPerfect Transfection Reagent only and cultured under normal oxygen tension. Fixation was carried out in ice-cold acetone for 10 minutes followed by air-drying and rehydration in PBS. The chamber slides were incubated for 1 h with the mouse monoclonal anti-human SERPINE1 antibody raised against amino acids 1 - 250 of the mature protein (1:50 dilution; TJA6, sc-59636, Santa Cruz Biotechnology Inc., Santa Cruz, CA, USA). For visualization the ZytoChem Plus HRP Polymer-Kit (Zytomed Systems, Berlin, Germany) and the DAB Substrate Kit High Contrast (Zytomed) were used. Counterstaining was accomplished with haematoxylin.

Immunohistochemistry on human kidney tissue was performed on tissue sections (1 - 2 μm) which were deparaffinised and treated with 3% H_2_O_2 _for 10 min. Following pre-treatment in a pressure cooker for retrieval of antigens, sections were incubated for 1 hour with the antibody against SERPINE1. Positive control for SERPINE1 was human placenta tissue and an isotype control monoclonal antibody (1:100 dilution, negative control for mouse IgG_1 _Ab-1, clone NCG01, DLN-05791, dianova, Hamburg, Germany) was used as negative control in lieu of the primary antibody.

## Results

### Hypoxia induced down-regulation of miRNA in primary fibroblasts

**Figure 1:**
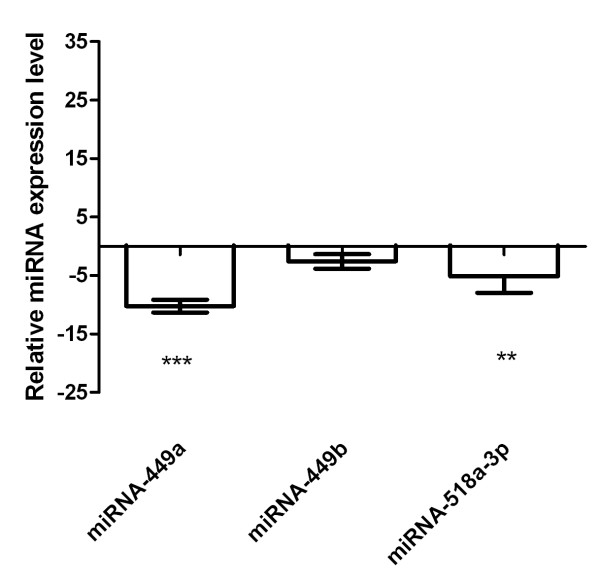
**MiRNA profiling and identification of candidates in fibroblasts under hypoxia**. MiRNA profiling of a total of 377 different miRNA species revealed down-regulation of miRNA-449a, -449b and -518a-3p when primary fibroblasts were cultured under hypoxic conditions (relative to RNU48). Shown are results from cell line F-18. *** = p < 0.001, ** = p < 0.01 (two independent experiments).

MicroRNA profiling determined that only 3 out of 377 miRNA subtypes were down-regulated in primary fibroblasts under hypoxic conditions compared with primary fibroblasts cultured under normal oxygen concentration: miRNA-449a (median -10.2, range -9.1 up to -11.3, p < 0.001), miRNA-449b (median -2.6, range -1.4 up to -3.8) and miRNA-518a-3p (median -5.1, range -2.3 up to -7.9, p < 0.01), Figure 1.

**Figure 2:**
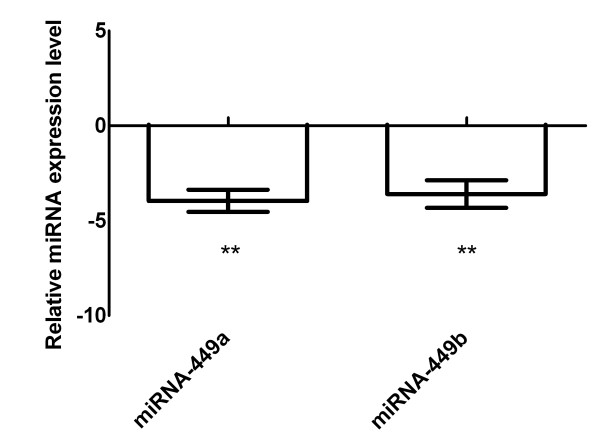
**Re-evaluation of miRNA-449a and miRNA-449b in fibroblasts**. Three independent experiments confirmed significant down-regulation under hypoxia. All calculations were performed relative to RNU48 in the cell line F-18. ** = p < 0.01.

Re-evaluation in an independent experimental setting confirmed down-regulation of miRNA-449a (median -4.0, range -2.9 up to -5.0, p < 0.01) and miRNA-449b (median -3.6, range -2.3 up to -4.9, p < 0.01), Figure 2.

### SERPINE1 is the most up-regulated gene in primary fibroblasts cultured under hypoxic conditions

**Figure 3:**
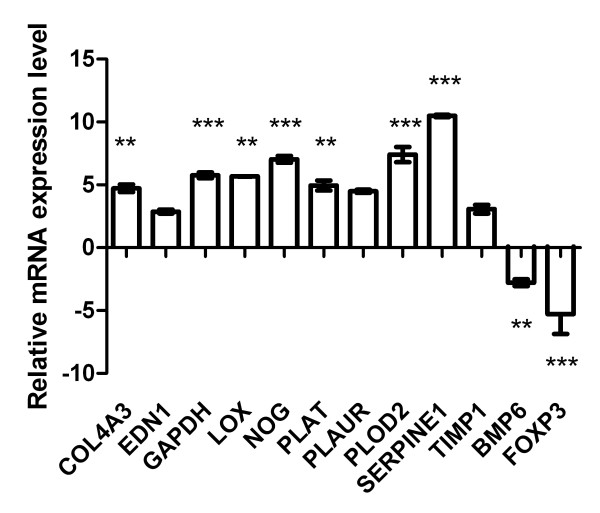
**Gene expression profiling in fibroblasts under hypoxia**. Primary human fibroblasts were analysed by LDA designed to determine fibrosis- and ECM-related gene expression. Depicted are the mean and range of gene expression in fibroblasts under hypoxia relative to reference gene POLR2A. Shown are results from cell line F-18. Among other factors induced in the hypoxic culture SERPINE1 mRNA was increased 10-fold. *** = p < 0.001, ** = p < 0.01 (two independent experiments).

Hypoxia induced overexpression of 10 target genes compared with gene expression profiling under normal oxygen conditions: Among those, SERPINE1 was significantly overexpressed by up to 10-fold (p < 0.001). Other inducible factors were COL4A3, LOX, PLAT, PLAUR, PLOD2, EDN1, GAPDH and inhibitors of matrix remodeling NOG and TIMP1. BMP6 and FOXP3 were down-regulated (Figure 3).

### Target prediction for miRNA-449a/b revealed SERPINE1 mRNA as a candidate

From the hypoxia-induced set of aberrantly expressed genes in fibroblasts (Figure 3) we screened for those potentially targeted by miRNA-449a/b. The TargetScan Database (http://microrna.sanger.ac.uk/targets/v5/, Wellcome Trust Sanger Institute) revealed SERPINE1 as a predicted target for both miRNA-449a and miRNA-449b (additional file 3 A + B).

**Figure 4:**
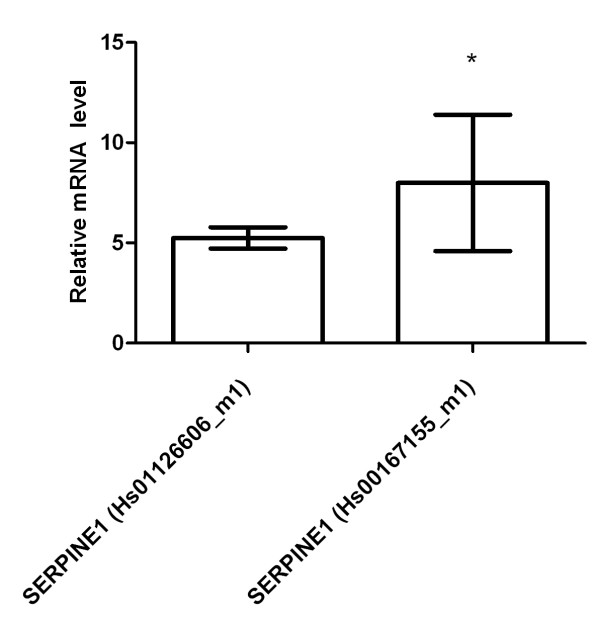
**Re-evaluation of SERPINE1 mRNA expression in fibroblasts under hypoxia**. Two different gene expression assays were applied to confirm SERPINE1 overexpression relative to POLR2A in fibroblasts cultured under hypoxia compared with fibroblasts cultured under normal oxygen conditions. Shown are results from cell line F-18. * = p < 0.05 (three independent experiments).

SERPINE1 overexpression in fibroblasts was then re-evaluated with 2 different commercially available gene expression assays following hypoxic culture in 3 independent experiments. Both assays confirmed SERPINE1 overexpression compared with normal oxygen conditions (median 5.3, 8.0, respectively), Figure 4.

### Hypoxia-driven inhibition and mimicking of miRNA-449 revealed direct targeting of SERPINE1 mRNA *in vitro*

**Figure 5:**
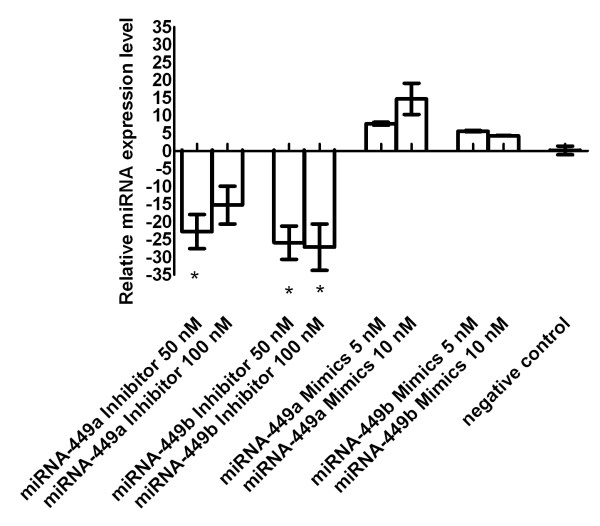
**MiRNA-449a/b expression under hypoxia**. Primary human fibroblasts were transfected with miRNA-449a/b inhibitor or miRNA-449a/b mimics and were cultured under hypoxia. Inhibition or mimicking strongly decreased or increased miRNA-449a/b expression, respectively. Depicted are calculations relative to reference gene RNU48 compared with non transfected cells cultured with hypoxia. Negative control means cells transfected with negative control siRNA only. Results are shown for cell line F-18 but were likewise demonstrable in M15D. * = p < 0.05 (three independent experiments).

Transfection followed by culture under hypoxic conditions induced increase and decrease of miRNA-449a/b by inhibition and mimicking, respectively. Inhibition showed down-regulation of miRNA-449a by up to 23-fold and 15-fold, for miRNA-449b by up to 26-fold and 27-fold (50 nM, 100 nM, respectively). Mimicking increased miRNA-449a by up to 8-fold and 15-fold, for miRNA-449b by up to 6-fold and 4-fold (5 nM, 10 nM, respectively), Figure 5.

**Figure 6:**
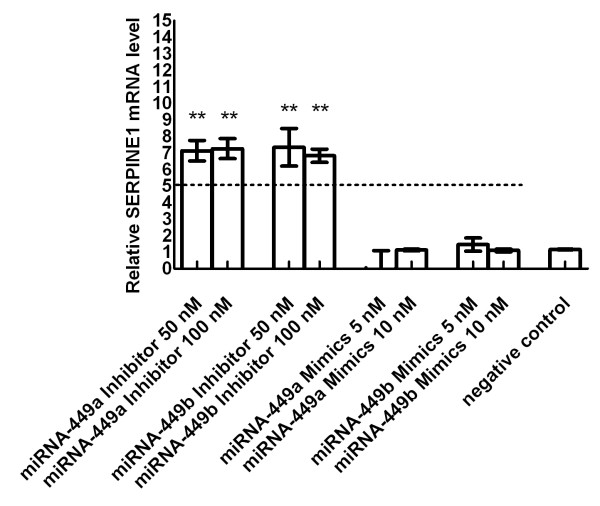
**SERPINE1 mRNA level in fibroblasts under hypoxia**. Transfection of miRNA-449a/b inhibitors and miRNA-449a/b mimics targeted SERPINE1 mRNA under hypoxic culture conditions. The mimicking of miRNA-449a/b reversed SERPINE1 mRNA to baseline level. Depicted are calculations relative to reference gene POLR2A compared with non-transfected cells cultured under hypoxia. Negative control means cells transfected with negative control siRNA only. SERPINE1 mRNA induction through hypoxia alone and without additional transfection of miRNA-449 inhibitors or miRNA-449 mimics is indicated by the dotted line (cell line F-18). ** = p < 0.01 (three independent experiments).

In a next step fibroblasts were studied for SERPINE1 mRNA expression under the similar experimental setting. When transfection studies were performed under hypoxia an increase of SERPINE1 mRNA by up to 8.0-fold (miRNA-449a inhibition: median 7.2, range 6.5 to 8.0; miRNA-449b inhibition: median 7.1, range 6.2 to 8.5) could be demonstrated compared with non-transfected cells cultured under hypoxia. Mimicking of miRNA-449a/b reversed SERPINE1 mRNA level thereby providing direct evidence for a targeting of SERPINE1 mRNA by miRNA-449a/b, Figure 6. The effects shown for SERPINE1 targeting by miRNA-449a/b were exclusively demonstrable in the hypoxic state of cell culture.

### Inhibition and mimicking of miRNA-449 affected SERPINE1 protein expression in fibroblasts

**Figure 7:**
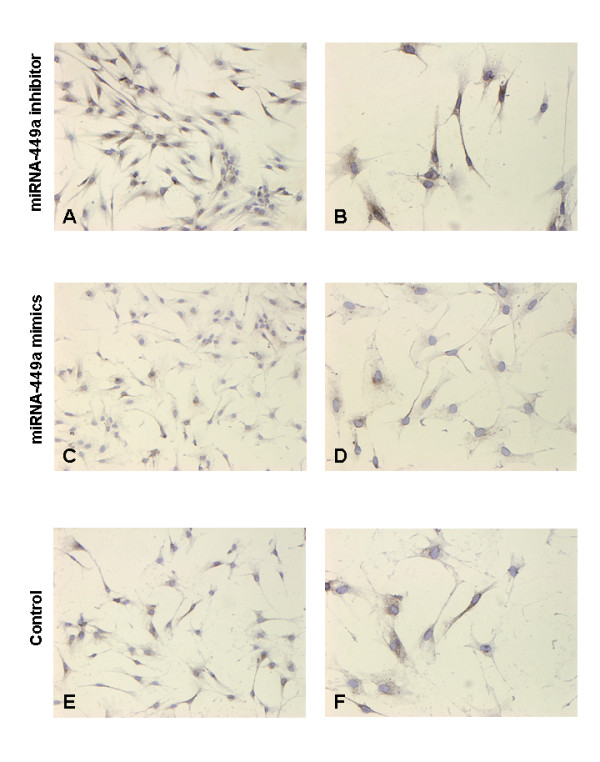
**Immunocytochemistry of primary human fibroblasts (F-18) transfected with miRNA-449a inhibitor or miRNA-449a mimics**. SERPINE1 strongly marked the fibroblasts transfected with miRNA-449a inhibitor (Figure 7 A + B) compared to miRNA-449a mimics which virtually blocks SERPINE1 protein expression (Figure 7 C + D). Non transfected primary human fibroblasts cultured under hypoxia showed SERPINE1 protein labeling in some cells (Figure 7 E + F). DAB immunostaining with mAb against SERPINE1 protein counterstained with haematoxylin. Magnification in Figure 7 A, C, E: 100×; Figure 7 B, D, F: 200×; brown: SERPINE1 positive fibroblasts; blue: nuclei (two independent experiments).

Following transfection with miRNA-449a inhibitor fibroblasts were cultured under hypoxic conditions and showed a strong induction of SERPINE1 protein (Figure 7A + B). By contrast, miRNA-449a mimics (Figure 7C + D) showed almost no SERPINE1 protein expression whereas control fibroblasts showed a faint SERPINE1 staining (Figure 7E + F).

### Chronic allograft remodeling showed the inverse expression of miRNA-449a/b and SERPINE1 expression

**Figure 8:**
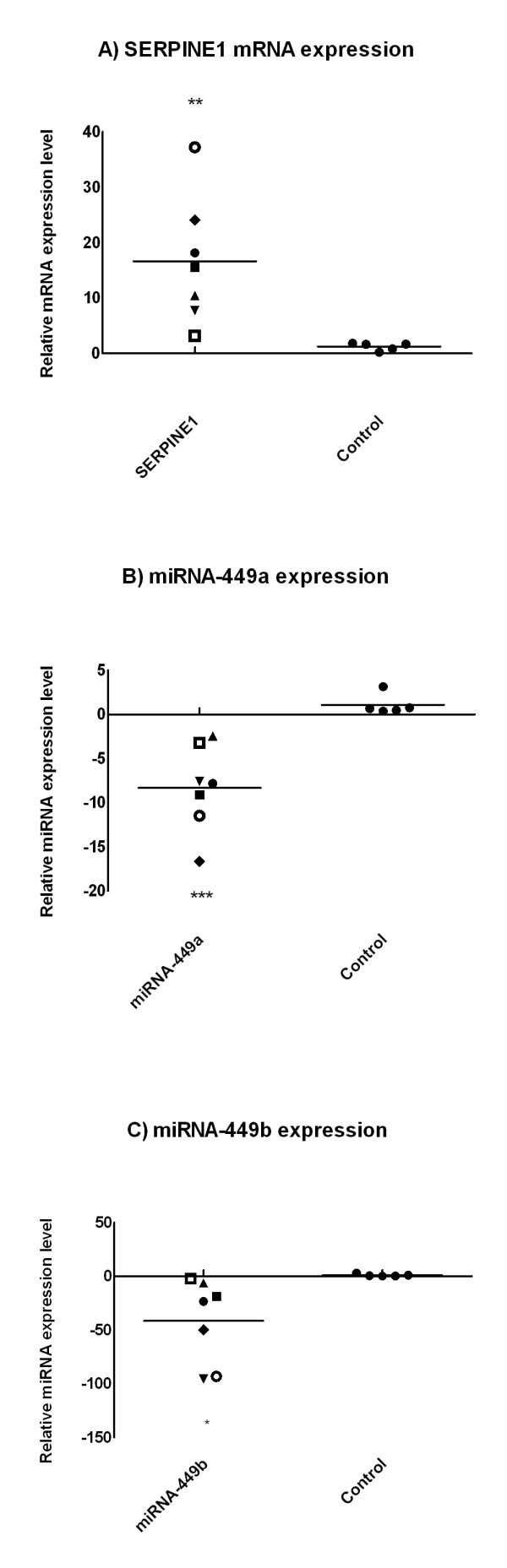
**Increased expression of SERPINE1 mRNA (A) and down-regulation of miRNA-449a/b (B + C) in human kidney transplants**. All cases showed an inverse mode of expression. Symbols represent the mean of 3 independent quantitative analyses of tissue blocks or biopsies as indicated in Table 1. The mean expression of SERPINE1 and miRNA-449a/b in the control kidney samples was arbitrarily set to 1. * = p < 0.05; ** = p < 0.01; *** = p < 0.001.

In human kidney transplants showing chronic allograft remodeling, miRNA-449a and miRNA-449b were down-regulated by up to 17.0-fold (median -7.8, range -2.5 to -16.6, p < 0.001) and 95.0-fold (median -23.3, range 2.3 to -95.4, p < 0.05), respectively (Figure 8 B + C). SERPINE1 mRNA expression in these specimens were increased by up to 37.0-fold (median 15.62, range 3.2 to 37.2, p < 0.01) when compared to control kidneys, Figure 8A.

**Figure 9:**
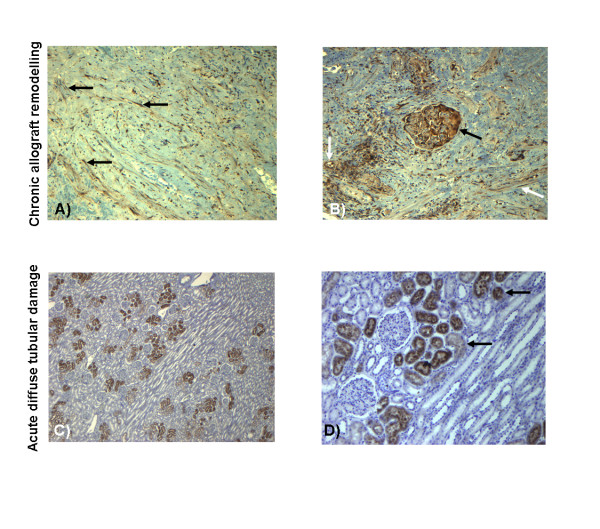
**Immunohistochemistry of kidney transplants showing chronic allograft remodelling revealed a strong labelling of SERPINE1 protein expression in areas of interstitial fibrosis and vascular remodelling (black arrows in A, white arrows in B)**. SERPINE1 positive cells were activated fibroblasts, endothelial cells and smooth muscle cells, Figure 9 A (magnification 100×). In not fully remodelled functional compartments glomerula and mesangial cells were prominently labeled (black arrow in B, magnification 200×). If present, scattered inflammatory cells stained SERPINE1 positive. In the control tissue derived from a kidney with acute tubular damage selected to ensure SERPINE1 expression only proximal tubuli (black arrows in D) were labelled in a considerable heterogenous pattern. Except for some smooth muscle cells SERPINE1 spares all other compartments (Figure 9C, D - magnification 25×, 100×, respectively). DAB immunostaining with mAb against SERPINE1 protein. Brown: SERPINE1 positive cells; blue: nuclei (two independent experiments).

SERPINE1 protein in kidney tissues was predominantly demonstrable in areas of interstitial fibrosis and vascular remodeling. Activated fibroblasts and smooth muscle cells were labeled. Glomerula and atrophic tubuli remodeled by connective tissue were also strongly stained (Figure 9A + B). Control kidney affected by acute diffuse tubular damage showed a heterogenous pattern with some strongly labeled proximal tubuli whereas other compartments were almost unlabelled. Some smooth muscle cells in smaller arterial vessels were also stained for SERPINE1 (Figure 9, C + D).

## Discussion

Uncontrolled tissue remodeling affects organ function, e.g. leads to dysfunction in transplanted organs. Acute tissue damage and wounding but also consecutive tissue repair and remodeling create a hypoxic environment and provide an appropriate condition for changes in gene expression due to hypoxia-sensitivity [20]. The fibroblast is the leading cell type involved in production of ECM following an adequate stimulus, i.e. one single activated fibroblast is able to induce thousands of pro-collagen molecules per minute [21]. It is the nature of tissue repair and remodeling that numerous factors and environmental conditions act in concert to guide the restoration process such as expression of hypoxia-inducible factors (HIF) and their target genes but also growth factors which induce a pro-fibrotic response [1]. Accordingly, at a glance it appears unlikely that a single aberration is predominantly involved in abnormal tissue remodeling. However, it has recently been shown by a decisive study that overexpression of miRNA-21 in fibroblasts is involved in myocardial fibrosis [14] suggesting that stress signaling could affect miRNA expression.

We found by miRNA profiling that only 3 out of 377 miRNA subtypes were down-regulated in primary fibroblasts when the stress factor hypoxia was tested. A single miRNA, i.e. the miRNA-184 was up-regulated (data not shown). The profiling showed no notable up-regulation of miRNA-21 in our approach. Previous work in cancer cell lines showed a much stronger affection by hypoxia with numerous miRNA being up- or down-regulated [22,23]. This difference can be explained by the fact that the basic miRNA profile in carcinoma cells is apparently much more aberrant than in normal cells and furthermore likely to be amplified by additional stress factors such as hypoxia.

We used only Pool A and therefore only 377 miRNA for profiling because trials in our lab using also Pool B showed very low numbers of detectable miRNA in different tissues and cell lines tested.

We selected the clustered miRNA subtypes miRNA-449a/b for confirmatory experiments in hypoxic fibroblasts because this decrease should lead to up-regulation of certain mRNA targets in this setting. Additionally, due to the immediate availability of low-density arrays designed to reveal important players in the pathogenesis of myelofibrosis [24] we performed gene expression profiling in hypoxic fibroblasts. Among other up-regulated factors involved in ECM synthesis and remodeling including HIF-1α targets LOX, EDN1 and GAPDH, SERPINE1 showed the strongest induction by up to 10-fold.

In the next step we asked if one of these hypoxia-inducible factors could be regulated by miRNA-449a/b. The miRBase Target Database (by using the link for TargetScan) listed SERPINE1 as a putative mRNA target of miRNA-449a/b.

We then tested the functional relationship between SERPINE1 and miRNA-449a/b by transfection of fibroblasts with miRNA-449 inhibitors or the respective mimics. SERPINE1 mRNA strongly increased through miRNA-449 inhibition while miRNA-449 mimics restored to baseline SERPINE1 mRNA levels. Of note, targeting of SERPINE1 mRNA by miRNA-449a/b was only demonstrable when hypoxia was present.

Though an increase of SERPINE1 could easily be explained by induction through HIF-1α the experiments using miRNA-449a/b mimics definitely support our proposed mechanism because these mimics induced a decrease of SERPINE1 mRNA level. Accordingly, labeling of SERPINE1 protein in these cell cultures confirmed a strong SERPINE1 protein expression when miRNA-449a was inhibited. When miRNA-449a was mimicked, almost no staining was demonstrable.

The knowledge on miRNA-449 action in humans is limited. In mice, the miRNA-449 was shown to be involved in the development of the choroid plexus [25]. In a model of post-ischemic regeneration in mice and patients with Duchenne muscular dystrophy miRNA-449 was induced and therefore referred to be a "regenerative miRNA" [26]. So it will be a matter of future efforts to test the dynamics of miRNA-449 expression during ischemia/hypoxia and post-hypoxia conditions, i.e. do miRNA-449a/b increase to normal levels when cells were exposed to normal oxygen levels?

We subsequently transferred the finding of inverse expression of miRNA-449/SERPINE1 to an *in vivo *setting, i.e. chronic allograft remodeling in kidney transplants. We assumed that initial tissue damage and ongoing tissue remodeling generate an environment with hypoxia, at least in certain areas, which affects miRNA-449 and SERPINE1 expression. Indeed, an inverse expression of miRNA-449a/b and SERPINE1 mRNA expression could be demonstrated in almost all patient samples. Apparently, a case-specific down-regulation of miRNA-449a/b expression must not necessarily correlate with the level of increased SERPINE1 mRNA. Consequently, morphological detection of the miRNA of interest should have been the next step. However, miRNA detection by e.g. in-situ-hybridization is more promising when the target of interest is expressed at considerable high levels what did not hold true for decreased miRNA-449. So we decided to look for the cellular source of SERPINE1 overexpression by immunohistochemistry. Because SERPINE1 mRNA and protein is undetectable in healthy kidney tissue [27] we selected a kidney with an acute diffuse tubular damage known to express SERPINE1 [28]. Kidney tubuli were labeled for SERPINE1 in a patchy pattern but clearly sparing all other compartments except for rarely stained smooth muscle cells of smaller vessels. By contrast, kidney allografts showed strong SERPINE1 labeling predominantly in activated fibroblasts and smooth muscle cells in areas of fibrotic and vascular remodeling, in atrophic tubuli and in the mesangium of glomeruli. Even though barely demonstrable, some inflammatory cells in the kidney transplants were also SERPINE1 positive.

SERPINE1 is known to be induced in kidney disease including kidney fibrosis and chronic allograft dysfunction [reviewed in [29]. SERPINE1 expression is inducible by numerous stimuli most notably by hypoxia and reactive oxygen species (ROS) themselves. However, in chronic hypoxia HIF-1α is no longer present [30] and thus can hardly be the inducer of SERPINE1 expression in a chronic remodeling process. TGFβ-1 can induce SERPINE1 expression but is inversely controlled by SERPINE1 via plasmin action [31]. Expression of tumor necrosis factor, driven by inflammation, can induce SERPINE1 [32] but apart from some scattered inflammatory cells inflammation was not the predominant feature in our samples.

Regulation of the SERPINE1 molecule is mainly achieved at the transcriptional level and the gene contains binding sites for several transcription factors such as Smads and HIFs [29]. However, the knowledge on post-transcriptional regulation of SERPINE1 mRNA is sparse. SERPINE1 mRNA contains several AU-rich destabilizing elements in the 3'-UTR suggesting a quick turnover. Interestingly, a recent study used a model of kidney fibrosis in rats to show that angiotensin II is able to stabilize SERPINE1 mRNA via the ubiquitous RNA-binding protein human-antigen R (HuR), [33].

We present a novel mechanism of post-transcriptional control of SERPINE1 where miRNA-449a/b lost the control over SERPINE1 mRNA through down-regulation under hypoxic conditions. If hypoxia-inducible factors themselves or hypoxia-triggered epigenetic mechanisms such as hypermethylation [30] interfere with miRNA-449a/b expression is a matter of ongoing investigations.

## Conclusions

We conclude that during remodeling of organs and tissues which lack the capability for self-renewal the normally tightly controlled and concerted action of different factors may be affected by aberrantly expressed miRNA species lastly leading to fibrosis development. As shown for hypoxia, decreased miRNA-449a/b contributes to increased SERPINE1 level due to impaired miRNA control of SERPINE1 mRNA fate *in vitro *and potentially likewise *in vivo*. This novel mechanism is demonstrable in chronic allograft remodeling of the kidney but probably also in other organ fibrosis and should be considered as a potential target for therapeutic intervention.

## Disclosure of competing interests

The authors declare that they have no competing interests.

## Authors' contributions

M.M. performed cell culture, real-time PCR, immunocytochemistry, preparation of the manuscript, interpretation of data; K.T. contributed to study design; K.H. interpretation of data; C.J. retrieval of control samples, preparation of RNA, interpretation of data; H.K. responsible for study design, data interpretation; O.B. responsible for study design, selection of cases, data interpretation, preparation of manuscript.

All authors have read and approved the final manuscript.

## Supplementary Material

Additional file 1 Tables showing LDA data of mRNA and miRNA expression. Click here for file

Additional file 2 Additional file lists target genes of custom-made LDA. Click here for file

Additional file 3 Target screening for miRNA-449a/b by using the TargetScan Database (http://microrna.sanger.ac.uk/targets/v5/, Wellcome Trust Sanger Institute) showed the binding of miRNA-449a (A) and miRNA-449b (B) at 7 consecutive positions within the 3'- UTR of the SERPINE1 mRNA. Click here for file
