# Supplementary material for: "Hypoxia-induced down-regulation of microRNA-449a/b impairs control over targeted SERPINE1 (PAI-1) mRNA - a mechanism involved in SERPINE1 (PAI-1) overexpression"
Source: J Transl Med. 2010 Apr 1;8:33. doi: 10.1186/1479-5876-8-33 (PMC2853517; doi:10.1186/1479-5876-8-33)
Supplement: Additional file 1 — Tables showing LDA data of mRNA and miRNA expression. [file 1479-5876-8-33-S1.DOC]

**Additional file 1 LDA mRNA**

| **Gene (supplier´s assay ID)** | **First array** | **Second array** | **mean** |
| --- | --- | --- | --- |
| BMP4-Hs00370078_m1 | -2,63 | -2,37 | -2,50 |
| **NOG-Hs00271352_s1** | **6,77** | **7,30** | **7,04** |
| BMP7-Hs00233476_m1 | ND | ND | ND |
| BMPR1B-Hs00176144_m1 | -2,60 | 1,35 | -1,15 |
| BMP1-Hs00241807_m1 | 1,08 | 1,23 | 1,15 |
| **BMP6-Hs01099594_m1** | **-2,51** | **-3,06** | **-2,78** |
| BMP2-Hs00154192_m1 | -1,96 | -1,90 | -1,93 |
| BMPR2-Hs00176148_m1 | 1,24 | 1,38 | 1,31 |
| SMAD3-Hs00706299_s1 | 1,10 | 1,08 | 1,09 |
| SMAD4-Hs00232068_m1 | -1,10 | -1,08 | -1,09 |
| **GAPDH-Hs99999905_m1** | **5,98** | **5,54** | **5,76** |
| TGFBR1-Hs00610318_m1 | 1,24 | 1,04 | 1,14 |
| SMAD5-Hs00195437_m1 | 1,03 | 1,17 | 1,10 |
| **SMAD1-Hs00195432_m1** | **-2,91** | **-2,80** | **-2,85** |
| TGFBRAP1-Hs00188614_m1 | -1,84 | -1,29 | -1,56 |
| MMP1-Hs00899658_m1 | 1,51 | 1,40 | 1,45 |
| MMP2-Hs00234422_m1 | 1,68 | 1,58 | 1,63 |
| MMP13-Hs00233992_m1 | -1,37 | -4,11 | -2,74 |
| MMP9-Hs00234579_m1 | ND | ND | ND |
| MMP14-Hs00237119_m1 | ND | ND | ND |
| THBS1-Hs00962908_m1 | ND | ND | ND |
| TGFBR2-Hs00234253_m1 | ND | ND | ND |
| TGFB2-Hs00234244_m1 | ND | ND | ND |
| TGFB3-Hs00234245_m1 | ND | ND | ND |
| COL1A2-Hs01028971_m1 | 1,46 | 1,40 | 1,43 |
| COL4A1-Hs01007469_m1 | 1,55 | 1,38 | 1,47 |
| MMP11-Hs00968295_m1 | 1,36 | 1,26 | 1,31 |
| COL18A1-Hs00181017_m1 | 1,41 | 1,38 | 1,40 |
| **LOX-Hs00184700_m1** | **5,68** | **5,66** | **5,67** |
| COL3A1-Hs00164103_m1 | 1,45 | 1,37 | 1,41 |
| COL4A2-Hs01098873_m1 | 1,53 | 1,45 | 1,49 |
| **COL4A3-Hs01022542_m1** | **5,02** | **4,43** | **4,73** |
| **FOXP3-Hs00203958_m1** | **-6,87** | **-3,71** | **-5,29** |
| CD9-Hs00233521_m1 | 2,00 | 1,87 | 1,94 |
| **PLOD2-Hs00168688_m1** | **8,02** | **6,80** | **7,41** |
| TNFRSF11B-Hs00171068_m1 | -1,97 | -2,06 | -2,01 |
| **TIMP1-Hs99999139_m1** | **3,41** | **2,72** | **3,07** |
| TIMP2-Hs00234278_m1 | 1,53 | 1,36 | 1,45 |
| **SERPINE1-Hs01126606_m1** | **10,58** | **10,38** | **10,48** |
| **PLAT-Hs00263492_m1** | **5,35** | **4,56** | **4,96** |
| **PLAUR-Hs00182181_m1** | **4,36** | **4,61** | **4,48** |
| **EDN1-Hs00174961_m1** | **3,03** | **2,71** | **2,87** |
| TGFB1-Hs00171257_m1 | ND | ND | ND |
| PTK2-Hs00178587_m1 | ND | ND | ND |
| IL6-Hs00985639_m1 | ND | ND | ND |
| GREM1-Hs00171951_m1 | ND | ND | ND |
|  |  |  |  |
| All target gene expression levels were related to POLR2A (Hs00172187_m1) as an endogenous control gene. Expression levels related to GUSB (Hs99999908_m1) were comparable.  ND = not detectable or not computable. | | | |

**Additional file 1 LDA miRNA**

| **miRNA (supplier´s assay ID)** | **First array** | **Second array** | **mean** |
| --- | --- | --- | --- |
| hsa-miR-184-4373113 | 33,51 | 17,38 | 25,45 |
| hsa-miR-449a-4373207 | -9,13 | -11,32 | -10,22 |
| hsa-miR-449b-4381011 | -3,83 | -1,37 | -2,60 |
| hsa-miR-518a-3p-4395508 | -7,96 | -2,31 | -5,13 |
|  |  |  |  |
| All miRNA expression levels were related to RNU48 (4373383) as a control miRNA. Other expression levels can be provided if requested. | | | |
