# Supplementary material for: "Hypoxia-induced down-regulation of microRNA-449a/b impairs control over targeted SERPINE1 (PAI-1) mRNA - a mechanism involved in SERPINE1 (PAI-1) overexpression"
Source: J Transl Med. 2010 Apr 1;8:33. doi: 10.1186/1479-5876-8-33 (PMC2853517; doi:10.1186/1479-5876-8-33)
Supplement: Additional file 2 — Additional file lists target genes of custom-made LDA. [file 1479-5876-8-33-S2.DOC]

| **#** | **Target genes** | **Synonym or full name** |
| --- | --- | --- |
| 1 | BMP1 | Bone morphogenetic protein 1 |
| 2 | BMP2 | Bone morphogenetic protein 2 |
| 3 | BMP4 | Bone morphogenetic protein 4 |
| 4 | BMP6 | Bone morphogenetic protein 6 |
| 5 | BMP7 | Bone morphogenetic protein 7 |
| 6 | BMPR1B | BMP receptor 1B |
| 7 | BMPR2 | BMP receptor 2 |
| 8 | NOG | Noggin |
| 9 | GREM1 | Gremlin |
| 10 | TGFB1 | Transforming growth factor β 1 |
| 11 | TGFB2 | Transforming growth factor β 2 |
| 12 | TGFB3 | Transforming growth factor β 3 |
| 13 | TGFBR1 | TGF β receptor 1 |
| 14 | TGFBR2 | TGF β receptor 2 |
| 15 | SDF1α | Stromal cell derived factor 1 α |
| 16 | SMAD1 | Mothers against decapentaplegic, Drosophila, homolog of, 1 |
| 17 | SMAD3 | Mothers against decapentaplegic, Drosophila, homolog of, 3 |
| 18 | SMAD4 | Mothers against decapentaplegic, Drosophila, homolog of, 4 |
| 19 | SMAD5 | Mothers against decapentaplegic, Drosophila, homolog of, 5 |
| 20 | EDN1 | Endothelin 1 |
| 21 | THBS1 | Thrombospondin 1 |
| 22 | MMP1 | Matrix metalloproteinase 1 |
| 23 | MMP2 | Matrix metalloproteinase 2 |
| 24 | MMP9 | Matrix metalloproteinase 9 |
| 25 | MMP11 | Matrix metalloproteinase 11 |
| 26 | MMP13 | Matrix metalloproteinase 13 |
| 27 | MMP14 | Matrix metalloproteinase 14 (membrane-type MMP 1) |
| 28 | TIMP1 | Tissue inhibitor of matrix metalloproteinase 1 |
| 29 | TIMP2 | Tissue inhibitor of matrix metalloproteinase 2 |
| 30 | IL6 | Interleukin 6 |
| 31 | PLAT | Plasminogen activator, tissue-type |
| 32 | PLAUR | Plasminogen activator, urokinase-type |
| 33 | SERPINE1 | Plasminogen activator inhibitor 1 |
| 34 | COL1A2 | Collagen type 1 α 2 |
| 35 | COL3A1 | Collagen type 3 α 1 |
| 36 | COL4A1 | Collagen type 4 α 1 |
| 37 | IL4 | Interleukin 4 |
| 38 | IL13 | Interleukin 13 |
| 39 | CCL5 | RANTES |
| 40 | LOX | Lysyl oxidase |
| 41 | PLOD2 | Lysyl hydroxylase 2 |
| 42 | TNFRSF11B | Osteoprotegerin |
| 43 | CXCR4 | Chemokine (C-X-C motif) receptor 4 |
| 44 | FOXP3 | Forkhead box P3 |
| 45 | PTK2 | Focal adhesion kinase |
|  | **Reference genes** |  |
| 46 | POLR2A | RNA-Polymerase 2 subunit A |
| 47 | GUSB | β-Glucuronidase |
| 48 | GAPDH | Glyceraldehyde-3-phosphate dehydrogenase |

**Additional file 2 - Overview of LDA target and control genes**
