# Supplementary figures and images for: "Hypoxia-induced down-regulation of microRNA-449a/b impairs control over targeted SERPINE1 (PAI-1) mRNA - a mechanism involved in SERPINE1 (PAI-1) overexpression"
Source: J Transl Med. 2010 Apr 1;8:33. doi: 10.1186/1479-5876-8-33 (PMC2853517; doi:10.1186/1479-5876-8-33)

**Additional file 3**


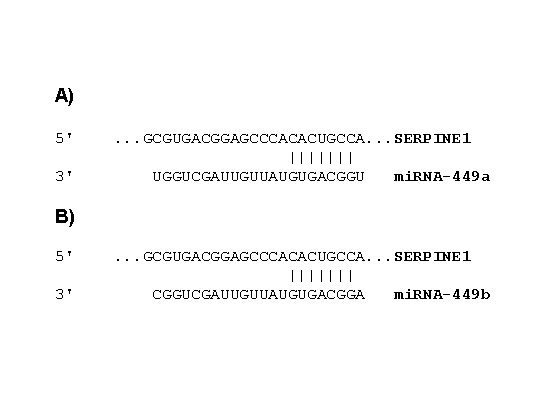

Supplement: Additional file 3 — Target screening for miRNA-449a/b by using the TargetScan Database (http://microrna.sanger.ac.uk/targets/v5/, Wellcome Trust Sanger Institute) showed the binding of miRNA-449a (A) and miRNA-449b (B) at 7 consecutive positions within the 3'- UTR of the SERPINE1 mRNA. [file 1479-5876-8-33-S3.DOC]
